# The Neck Circumference-to-Shoulder Width Ratio as a Novel Anthropometric Indicator for Dyslipidemia Screening in Adults at a Tertiary Care Hospital: A Cross-Sectional Study

**DOI:** 10.7759/cureus.90344

**Published:** 2025-08-17

**Authors:** Arun Kumar Sai A, Vinoth Kumar Kalidoss, Sarah Ramamurthy, Yamini Marimuthu

**Affiliations:** 1 Community and Family Medicine, All India Institute of Medical Sciences, Mangalagiri, Mangalagiri, IND; 2 Anatomy, All India Institute of Medical Sciences, Madurai, Madurai, IND; 3 Preventive Medicine, Maulana Azad Medical College, New Delhi, IND

**Keywords:** dyslipidemia, neck circumference, non-communicable diseases, obesity, shoulder width

## Abstract

Introduction: Dyslipidemia has become a significant health problem, often affecting individuals' psychosocial and physical health. Various anthropometric parameters were used for the screening of dyslipidemia. This study evaluates the reliability of the neck circumference (NC) to shoulder width (SW) ratio as a novel anthropometric indicator in the screening of obesity, correlating with BMI, waist-to-hip ratio, and lipid profile.

Materials and methods: This is a cross-sectional study conducted at the outpatient department of Community and Family Medicine at the All India Institute of Medical Sciences, Mangalagiri. Patients aged more than 18 years who underwent lipid profile investigation were included, and persons with neck swelling or injury, shoulder injury, pregnant women, hormonal disorders, congenital anomalies, and those post-radiotherapy were excluded from the study. Anthropometric measurements were conducted according to standard guidelines. The correlation between the NC/SW ratio and the body mass index, waist-to-hip ratio, and lipid profile parameters (total cholesterol, high-density lipoprotein (HDL), and non-HDL) was assessed using the Pearson correlation coefficient. A p-value less than 0.05 was considered statistically significant. Data analysis was performed using SPSS Statistics version 26.0 (IBM Corp., Released 2019. IBM SPSS Statistics for Windows, Version 26.0. Armonk, NY: IBM Corp.).

Results: Approximately 98 participants with complete data were included for the analysis, of which 50% belonged to the 41-60 years age group, and 50% were males. Diabetes and hypertension were present in 27% and 35% of the study participants, respectively. The mean (SD) NC, SW, and NC/SW ratio of the study participants were 36.6 (3.6), 41.7 (3.8), and 0.9 (0.1), respectively. The majority of participants were obese (63%), and 28% were overweight. Among the study participants, around 87.8% had at least one abnormal lipid profile. NC had a significant positive correlation with total cholesterol, triglycerides, and LDL level, and a negative correlation with HDL level. The NC/SW ratio showed a positive correlation with total cholesterol and triglyceride levels.

Conclusions: NC was significantly correlated with BMI, waist circumference, hip circumference, and lipid profile. The NC/SW ratio also exhibited a correlation with BMI. The NC and NC/SW ratio can be used for the screening of dyslipidemia.

## Introduction

Dyslipidemia has become a significant health problem, often affecting individuals' psychosocial and physical health. Individuals with morbid obesity are at risk of developing comorbidities such as diabetes, coronary vascular diseases, stroke, fatty liver, and depression [1]. Dyslipidemia is biochemically defined as increased total or low-density lipoprotein (LDL) cholesterol levels or low levels of high-density lipoprotein (HDL) cholesterol [2]. The ICMR-INDIAAB study showed that around 79% of the Indian population has at least one abnormal lipid parameter. Among the various lipid profile parameters, low HDL cholesterol (72.3%) was the most common abnormality, followed by hypertriglyceridemia (29.5%) [3]. A recent study estimated that obesity will triple among Indian adults by 2040. The study showed that the prevalence of obesity is estimated to be 14.0% among women and 9.5% (5.4-13.3%) in men by 2040 [4].

Halting the rise in diabetes and obesity is one of the nine goals that must be achieved in the WHO Global NCD Action Plan 2013-2020 [5]. It is essential to identify the risk factors and screen for obesity as early as possible to prevent the subsequent sequelae of morbidities. Obesity and dyslipidemia are significant factors in various cardiovascular risk prediction charts, like the WHO's cardiovascular risk chart. Dyslipidemia can be diagnosed with the help of CT and MRI with the greatest accuracy for determining visceral fat, though they act as a gold standard for diagnosis [6]. They have very poor patient compliance due to higher expenses; the same concern applies to serum analysis for dyslipidemia in low-income families.

A much more affordable alternative for diagnosis is anthropometric indicators like the waist-to-hip ratio and BMI. The BMI, as such, cannot be considered a complete indicator of dyslipidemia in muscular individuals. It cannot differentiate between lean muscle mass and fat; it does not consider the variation in fat distribution, so other parameters like waist-to-hip ratio are needed [7]. The government of India's National Program for Prevention and Control of Non-communicable Diseases program uses community-based screening for non-communicable diseases. It measures waist circumference (WC) instead of BMI, as measuring BMI in the field requires more resources, such as a standard weighing scale and stadiometer. Therefore, a cost-effective strategy will be measuring a circumference anthropometry parameter by frontline health workers for estimating non-communicable diseases [8].

The waist-to-hip ratio also has limitations, as it is influenced by breath movements, postprandial abdominal distension, and pregnancy [9,10]. A study concluded that the waist-to-hip ratio in children and adolescents is not a good measure of fat deposition. Sometimes the measurement procedure is not amiable in Indian rural settings and among the elderly and disabled [9].

Neck circumference (NC) has already been considered a reliable indicator of dyslipidemia through many research analyses correlating metabolic tests like lipid profile (HDL, non-HDL, total cholesterol) [10-12]. NC varies from lean to muscular, leading to discrepancies. This necessitates additional parameters with NC to increase the reliability and accuracy of the anthropometric indicator, where shoulder width (SW) can be considered an additional parameter. However, studies done so far on this approach are limited. This study evaluates the reliability of the NC/SW ratio as a novel anthropometric indicator in the screening of obesity in correlation to BMI, waist-to-hip ratio, and lipid profile. With this background, this study aims to determine the correlation between the NC/SW ratio and the lipid profile, body mass index, and waist-to-hip ratio of the adult population attending tertiary care hospitals.

## Materials and methods

This is a cross-sectional study conducted at the outpatient department of Community and Family Medicine at the All India Institute of Medical Sciences, Mangalagiri. Patients aged more than 18 years who attended the outpatient department for lipid profile investigation and were willing to participate in the study from July 2022 to September 2022 were included in the study consecutively. Persons with neck swelling or injury, shoulder injury, pregnant women, hormonal disorders, congenital anomalies, and those post-radiotherapy were excluded from the study.

Expecting a correlation coefficient of more than 0.5 between the NC/SW ratio and total cholesterol, with a 5% alpha error and 90% power, the sample size was calculated to be 73 using nMaster software (Department of Biostatistics at Christian Medical College, Vellore, India). After adjusting for a 30% non-response rate and missing data, the final sample size was 95.

After obtaining written consent from the participants, a structured proforma was used to collect data. The first part of the proforma contains details about basic socio-demographics and clinical history. Anthropometric measurements were conducted according to standard guidelines [13]. Lipid profile results were later collected from the hospital information management system using the patient identification number.

Operational definitions

NC was measured with a calibrated plastic tape in the midline of the neck, between the mid-cervical spine and mid-anterior neck, within 1 mm. Regarding SW, it is measured with a calibrated plastic tape between the lateral borders of the acromion processes (the two bones at the ends of the shoulders).

According to the National Cholesterol Education Programme, Adult Treatment Panel III guidelines, anyone who meets any of the following criteria (serum total cholesterol levels ≥200 mg/dl, serum triglyceride levels ≥150 mg/dl, HDL cholesterol levels <40 mg/dl, or LDL cholesterol levels ≥130 mg/dl) was considered dyslipidemia [14].

Statistical analysis

The categorical variables, such as gender, history of diabetes, and hypertension, were summarized as frequency and proportion. Continuous variables like age, BMI, NC, SW, and total cholesterol were summarized as mean and standard deviation based on the normality of the data using the Shapiro-Wilk test. The correlation between the NC/SW ratio and the body mass index, waist-to-hip ratio, and lipid profile parameters (total cholesterol, HDL, and non-HDL) was assessed using the Pearson correlation coefficient. A p-value less than 0.05 was considered statistically significant. Data analysis was performed using SPSS Statistics version 26.0 (IBM Corp., Released 2019. IBM SPSS Statistics for Windows, Version 26.0. Armonk, NY: IBM Corp.).

Ethical considerations

The study was approved by the Institutional Ethical Committee of All India Institute of Medical Sciences, Mangalagiri (AIIMS/MG//IEC/2022-23/175) before starting data collection. After obtaining written consent from the participants, a structured proforma was used to collect data.

## Results

Approximately 100 participants were enrolled in the study, and 98 participants with complete data were included in the analysis. Table 1 presents the basic demographic and clinical profiles of the study participants. The majority of participants belonged to the 41-60 years age group (50%), followed by the 20-40 years age group (30.6%). Half of the participants were male, 27% were known to have diabetes mellitus, and approximately 35% had hypertension. The anthropometric profile of the study participants is also shown in Table 1. The mean (SD) weight and height of the participants were 72.7 (14.1) kg and 158.4 (9.6) cm, respectively. The mean (SD) BMI of the participants was 29 (5.2) kg/m². The mean (SD) NC, SW, and NC/SW ratio of the participants were 36.6 (3.6) cm, 41.7 (3.8) cm, and 0.9 (0.1), respectively.

**Table 1:** Characteristics of the study participants (N=98) BMI: body mass index, WC: waist circumference, HC: hip circumference, NC: neck circumference, SW: shoulder width, HDL: high-density lipoprotein, LDL: low-density lipoprotein, HbA1c: glycosylated hemoglobin, SD: standard deviation, H/o: history of, DM: diabetes mellitus, HTN: hypertension, BMI: body mass index, FBS: fasting blood sugar, PPBS: postprandial blood sugar, cat: category

Variable	Categories	N(%)/mean (SD)
Age group	20-40 years	30 (30.6)
	41-60 years	49 (50.0)
	>60 years	19 (19.4)
Gender	Female	49 (50.0)
	Male	49 (50.0)
H/o DM	0	72 (73.5)
	1	26 (26.5)
H/o HTN	0	64 (65.3)
	1	34 (34.7)
Weight (Kg)		72.7 ± 14.1
Height (cm)		158.4 ± 9.6
BMI		29.0 ± 5.2
WC (cm)		92.1 ± 8.7
HC (cm)		107.4 ± 12.1
WC/HC		0.9 ± 0.1
NC (cm)		36.6 ± 3.6
SW (cm)		41.7 ± 3.8
NC/SW		0.9 ± 0.1
Total cholesterol	Normal	69 (70.4)
	>200mg/dl	29 (29.6)
Triglycerides	Normal	48 (49.0)
	>150	50 (51.0)
HDL cat	Normal	60 (61.2)
	<40 mg/dl	38 (38.8)
LDL cat	Normal	82 (83.7)
	130 mg/dl	16 (16.3)
FBS binary	<126	56 (57.1)
	≥126	42 (42.9)
PPBS cat	<200	56 (58.1)
	≥200	42 (42.9)
HbA1c	<6.5	40 (40.8)
	≥6.5	58 (59.2)

Among the lipid profiles, the major abnormality was found in triglyceride levels (51%). Approximately 29% of participants had high total cholesterol, and 39% had low HDL. Around 60% of participants had high HbA1c levels, and 43% had high fasting and postprandial sugars. Notably, 87.8% of the study participants had at least one abnormal lipid profile.

The association between various anthropometric parameters and total cholesterol and triglyceride levels among participants is depicted in Table 2. For total cholesterol, individuals with levels over 200 mg/dL had slightly higher mean weights (74.2 kg vs. 72.1 kg), but the difference was not statistically significant (p=0.496). Height, BMI, waist circumference (WC), hip circumference (HC), WC/HC ratio, and NC/SW ratio also showed no significant differences between the two groups, with p-values of 0.787, 0.527, 0.411, 0.966, 0.334, and 0.384, respectively. However, NC was marginally higher in individuals with elevated cholesterol (37.5 cm vs. 36.2 cm) with a p-value of 0.094. In terms of triglycerides, individuals with higher triglyceride levels had a significantly greater NC (37.4 cm vs. 35.8 cm) and a higher NC/SW ratio (0.89 vs. 0.86), with p-values of 0.029 and 0.042, respectively. Other parameters such as weight, height, BMI, WC, HC, SW, and WC/HC ratio did not show statistically significant differences, as indicated by their p-values.

**Table 2:** Comparison anthropometry and total cholesterol and triglyceride levels of study participants BMI: body mass index, WC: waist circumference, HC: hip circumference, NC: neck circumference, SW: shoulder width, SD: standard deviation

Parameter	Total cholesterol		
	Normal	>200 mg/dl	p-value
	Mean ± SD	Mean ± SD	
Weight	72.1 ± 15.0	74.2 ± 11.7	0.496
Height	158.2 ± 10.1	158.8 ± 11.7	0.787
BMI	28.8 ± 5.3	29.5 ±	0.527
WC	91.6 ± 9.0	93.2 ± 8.0	0.411
CH	107.3 ± 12.1	107.4 ± 12.4	0.966
WC/HC	0.9 ± 0.1	0.9 ± 0.1	0.334
NC	36.2 ± 3.5	37.5 ± 3.6	0.094
SW	41.5 ± 3.9	42.3 ± 3.5	0.354
NC/SW ratio	0.9 ± 0.1	0.9 ± 0.1	0.384
	Triglyceride		
	Normal	>150 mg/dl	
Weight	71.2 ± 12.2	74.2 ± 15.7	0.284
Height	157.8 ± 8.9	159.0 ± 10.4	0.518
BMI	28.8 ± 5.6	29.2 ± 4.8	0.672
WC	91.6 ± 8.5	92.5 ± 8.9	0.605
HC	106.9 ± 13.4	107.9 ± 10.8	0.684
WC/HC	0.9 ± 0.1	0.9 ± 0.1	0.843
NC	35.8 ± 3.3	37.4 ± 3.7	0.029
SW	41.5 ± 3.4	42.0 ± 4.2	0.533
NC/SW ratio	0.86 ± 0.05	0.89 ± 0.07	0.042

The association between various anthropometric parameters and HDL and LDL cholesterol levels among participants is depicted in Table 3. Participants with HDL levels below 40 mg/dL had a higher mean weight (74.8 kg vs. 71.5 kg) and height (160.1 cm vs. 157.4 cm) compared to those with normal HDL levels, but these differences were not statistically significant (p=0.260 and p=0.183). The BMI, WC, HC, WC/HC ratio, and NC/SW ratio showed no significant differences between the two HDL groups (p>0.05). However, NC and SW were significantly larger in the low HDL group, with mean values of 37.7 cm and 43.0 cm, respectively, compared to 35.9 cm and 40.9 cm in the normal HDL group (p=0.019 and p=0.009).

**Table 3:** Comparison anthropometry and level of HDL and LDL of study participants BMI: body mass index, WC: waist circumference, HC: hip circumference, NC: neck circumference, SW: shoulder width, HDL: high-density lipoprotein, LDL: low-density lipoprotein, SD: standard deviation

Parameter	HDL level		
	Normal	<40 mg/dl	p-value
	Mean ± SD	Mean ± SD	
Weight	71.5 ± 13.2	74.8 ± 15.4	0.260
Height	157.4 ± 10.0	160.1 ± 9.0	0.183
BMI	28.9 ± 5.3	29.1 ± 5.2	0.862
WC	91.7 ± 9.1	92.6 ± 8.0	0.602
HC	108.0 ± 12.2	106.4 ± 12.2	0.541
WC/HC	0.9 ± 0.1	0.9 ± 0.1	0.129
NC	35.9 ± 3.6	37.7 ± 3.3	0.019
SW	40.9 ± 3.4	43.0 ± 4.1	0.009
NC/SW ratio	0.88 ± 0.08	0.88 ± 0.07	0.987
	LDL levels		
	Normal	>130 mg/dl	
Weight	71.5 ± 13.2	74.8 ± 15.4	0.034
Height	157.4 ± 10.0	160.1 ± 9.0	0.667
BMI	28.9 ± 5.3	29.1 ± 5.2	0.029
WC	91.7 ± 9.1	92.6 ± 8.0	0.067
HC	108.0 ± 12.2	106.4 ± 12.2	0.559
WC/HC	0.9 ± 0.1	0.9 ± 0.1	0.127
NC	35.9 ± 3.6	37.7 ± 3.3	<0.001
SW	40.9 ± 3.4	43.0 ± 4.1	0.057
NC/SW ratio	0.87 ± 0.07	0.91 ± 0.07	0.052

For LDL levels, individuals with higher LDL showed significantly greater mean weight (74.8 kg vs. 71.5 kg) and BMI (29.1 vs. 28.9) with p=0.034 and p=0.029, respectively. NC was significantly larger in the high LDL group (37.7 cm vs. 35.9 cm, p<0.001). Other parameters, such as WC, HC, SW, WC/HC ratio, and NC/SW ratio, showed no statistically significant differences between the two LDL groups.

Table 4 and Table 5 depict the examined correlations between anthropometric parameters and lipid profiles among participants. NC showed significant positive correlations with weight (r=0.784, p<0.01), height (r=0.448, p<0.01), BMI (r=0.537, p<0.01), WC (r=0.570, p<0.01), and HC (r=0.354, p<0.01). Additionally, NC was positively correlated with SW (r=0.190, p=0.05) but did not have a significant correlation with the WC/HC ratio. SW demonstrated significant positive correlations with weight (r=0.601, p<0.01), height (r=0.545, p<0.01), BMI (r=0.263, p<0.01), and WC (r=0.410, p<0.01). It also showed a positive correlation with the WC/HC ratio (r=0.229, p=0.05) and was strongly correlated with NC (r=0.190, p=0.05). The NC/SW ratio was positively correlated with weight (r=0.279, p<0.01), BMI (r=0.346, p<0.01), WC (r=0.239, p=0.05), and HC (r=0.227, p=0.05), but showed no significant correlation with height or the WC/HC ratio.

**Table 4:** Correlation of anthropometry parameters of study participants ** Correlation is significant at the 0.01 level. * Correlation is significant at the 0.05 level. BMI: body mass index, WC: waist circumference, HC: hip circumference, NC: neck circumference, SW: shoulder width

Parameters	Height	BMI	WC	HC	WC/HC	NC	SW	NC/SW ratio
Weight	0.438^**^	0.765^**^	.714^**^	.655^**^	-0.050	.784^**^	.601^**^	.279^**^
Height	1	0.234^*^	0.196	.205^*^	.499^**^	.448^**^	.545^**^	-0.050
BMI		1	.638^**^	.855^**^	-.400^**^	.537^**^	.263^**^	.346^**^
WC			1	.688^**^	.223^*^	.570^**^	.410^**^	.239^*^
HC				1	-.548^**^	.354^**^	0.176	.227^*^
WC/HC					1	0.190	.229^*^	-0.012

**Table 5:** Correlation of anthropometry parameters and lipid profile among study participants ** Correlation is significant at the 0.01 level. * Correlation is significant at the 0.05 level. BMI: body mass index, WC: waist circumference, HC: hip circumference, NC: neck circumference, SW: shoulder width, TC: total cholesterol, TG: triglycerides, HDL: high-density lipoprotein, LDL: low-density lipoprotein

Parameters	TC	TG	HDL	LDL
Weight	0.221^*^	0.211^*^	-0.127	0.132
Height	0.000	0.193	-0.190	-0.009
BMI	0.242^*^	0.064	0.016	0.169
WC	0.092	0.075	-0.094	0.017
HC	0.073	-0.005	0.098	-0.002
WC/HC	0.025	0.073	-0.239^*^	0.053
NC	0.293^**^	0.327^**^	-0.246^*^	0.217^*^
SW	0.035	0.172	-.207^*^	0.052
NC/SW ratio	0.310^**^	0.221^*^	-0.079	0.186

Notably, NC showed a significant positive correlation with total cholesterol (r=0.293, p<0.01), triglycerides (r=0.327, p<0.01), and LDL (r=0.217, p<0.05), while being negatively correlated with HDL (r=-0.246, p<0.05). The NC/SW ratio also had significant positive correlations with total cholesterol (r=0.310, p<0.01) and triglycerides (r=0.221, p<0.05). Weight was positively correlated with total cholesterol (r=0.221, p<0.05) and triglycerides (r=0.211, p<0.05). BMI showed a significant positive correlation with total cholesterol (r=0.242, p<0.05), while the WC/HC ratio had a significant negative correlation with HDL (r=-0.239, p<0.05).

## Discussion

The study results revealed a prevalence of obesity among men and women of 53% and 74%, respectively, which is significantly higher than the 4% and 6.4% reported among men and women in the National Family Health Survey 5 report [15]. These discrepancies in prevalence rates may be attributed to the differences in patient profiles, as our study recruited participants from a hospital setting. Our study found that 87.8% of participants had at least one abnormal lipid profile parameter, compared to 79% reported in the ICMR-INDIAB study [3]. Notably, our study showed that the most common lipid abnormalities were hypertriglyceridemia (high triglycerides), followed by low HDL cholesterol. In contrast, the ICMR-INDIAB study [3] reported low HDL cholesterol as the most common abnormality, followed by high triglycerides.

The current study showed the mean NC of the participants was 36.6 cm, and the NC of males and females was 38.1 cm and 35.1 cm, respectively. These results were similar to another study done among the Indian population [11] that showed the mean NC of all participants was 34.3 cm, and the NC of males and females was 34.9 cm and 33.65 cm, respectively. Our results were also similar to a study done among the Thai population [16] that showed the mean NC of all participants was 35.7 cm, and the NC of males and females was 38.4 cm and 33.9 cm, respectively. Another study done among the Chinese [17] showed that the mean NC of males and females was 38.4 cm and 35.4 cm, respectively. It also suggests that an NC of ≥37 cm for men and ≥35 cm for women was the best cut-off point to determine central obesity. Our study results showed the mean NC of the participants with obesity was 38.4 cm. The current study showed the mean (SD) SW of males and females were 43.6 cm and 39.9 cm, which is similar to a study by Igiri et al. [18]. It was found that the SW of males (37.9 cm) was statistically higher than that of females (34.8 cm) [18].

Our study revealed a Pearson correlation coefficient of 0.537 between NC and BMI and 0.570 between NC and WC. The study by Preis et al. [19] showed that the Pearson correlation coefficients between NC and BMI and between NC and WC were 0.79 and 0.75 for males and 0.78 and 0.80 for females, respectively.

The current study demonstrated a moderate correlation between NC and HDL cholesterol levels (r=-0.246), consistent with previous research (r=-0.29) [19]. In contrast to the study done by Zhang et al. [20] among the Chinese population and an analysis of the Framingham Heart Study [19], which found no correlation between NC and total cholesterol (r=0.03 and r=0.09, respectively), our study revealed a weak positive correlation (r=0.293). The association of NC with triglycerides (r=0.327) and LDL (r=0.217) in our study aligns with the findings of Zhang et al. [20] and Preis et al. [19]. A systematic review and meta-analysis by Shokri-Mashhadi et al. [21] reported pooled correlations of NC with HDL (r=-0.27) and triglycerides (r=0.34), similar to our results.

Recent epidemiological studies [22,23] have consistently shown strong correlations between NC and WC, as well as BMI, highlighting its utility as a marker for cardiovascular disease risk factors. Our study suggests that NC can be a valuable tool for identifying individuals at high risk of diseases related to glucose and lipid metabolism, as well as other components of metabolic syndrome.

The findings of our study, building on the work of Igiri et al. [18], which demonstrated a correlation between NC and SW in both males and females, reveal a significant association between the NC/SW ratio and key biomarkers of metabolic syndrome, including total cholesterol (r=0.310) and triglycerides (r=0.221). These results suggest that the NC/SW ratio may serve as a valuable adjunct tool for identifying individuals at high risk of developing diseases linked to glucose and lipid metabolism. Further research is warranted to explore the utility of the NC/SW ratio as a potential risk factor for metabolic syndrome and its components, offering a novel avenue for early detection and prevention strategies.

There are a few limitations to the study. First, as a cross-sectional study, it cannot establish causal relationships. Second, the sample size is too small to make adjustments for confounders, and the generalizability of the findings should be interpreted with caution, as the study was conducted in a single tertiary care center in South India, which may not be representative of other populations. Nevertheless, this study provides valuable insights into the potential utility of the NC/SW ratio as a marker for dyslipidemia and obesity, with anthropometric measurements taken following standard guidelines to ensure data consistency and reliability.

## Conclusions

Approximately one-third of the study participants were overweight, while two-thirds were obese. NC showed a significant correlation with BMI, WC, and HC. Additionally, the NC/SW ratio was also found to be correlated with BMI. Notably, NC was correlated with all parameters of the lipid profile. Our study investigated the potential utility of NC and the NC/SW ratio as screening tools for obesity and dyslipidemia. To establish causality between the NC/SW ratio and lipid profile or other health outcomes, longitudinal studies are necessary. Further exploration of the relationship between the NC/SW ratio and metabolic health may reveal new opportunities for personalized interventions and targeted therapies for non-communicable diseases.
